# Systems approaches to investigate the role of NF-κB signaling in aging

**DOI:** 10.1042/BCJ20210547

**Published:** 2022-01-28

**Authors:** Masatoshi Haga, Mariko Okada

**Affiliations:** 1 Laboratory for Cell Systems, Institute for Protein Research, Osaka University, Suita, Osaka 565-0871, Japan; 2 Basic Research Development Division, ROHTO Pharmaceutical Co., Ltd., Ikuno-ku, Osaka 544-8666, Japan; 3 Center for Drug Design and Research, National Institutes of Biomedical Innovation, Health and Nutrition, Ibaraki, Osaka 567-0085, Japan

**Keywords:** aging, mathematical model, nuclear factor kappa B, systems biology, transcription factors

## Abstract

The nuclear factor-κB (NF-κB) signaling pathway is one of the most well-studied pathways related to inflammation, and its involvement in aging has attracted considerable attention. As aging is a complex phenomenon and is the result of a multi-step process, the involvement of the NF-κB pathway in aging remains unclear. To elucidate the role of NF-κB in the regulation of aging, different systems biology approaches have been employed. A multi-omics data-driven approach can be used to interpret and clarify unknown mechanisms but cannot generate mechanistic regulatory structures alone. In contrast, combining this approach with a mathematical modeling approach can identify the mechanistics of the phenomena of interest. The development of single-cell technologies has also helped clarify the heterogeneity of the NF-κB response and underlying mechanisms. Here, we review advances in the understanding of the regulation of aging by NF-κB by focusing on omics approaches, single-cell analysis, and mathematical modeling of the NF-κB network.

## Introduction

The transcription factor nuclear factor-κB (NF-κB) is responsible for regulating genes associated with inflammation [1]. NF-κB is linked not only to diseases such as cancer but also to the aging process [2]. Aging is an unavoidable time-dependent decline in physiological organ function. By disrupting the homeostasis of health, aging rapidly increases the risk of death from cancer, diabetes, or heart disease [3,4].

As we age, our bodies accumulate senescent cells [5,6]. Notably, the activation of NF-κB signaling promotes cell senescence [7]; NF-κB activation has been known to shorten the lifespan of fruit flies [8] and mice [9,10]. Furthermore, an increase in NF-κB DNA-binding activity has been reported in dermal fibroblasts and renal tissues derived from elderly individuals [11,12]. Therefore, a comprehensive understanding and control of the NF-κB system may shed light on how to prevent age-related diseases and prolong lifespan.

NF-κB is the primary regulator of senescence-associated secretory phenotype (SASP), which consists of inflammatory cytokines (interleukin [IL]-6 and IL-8), proteases (matrix metalloproteinases), chemokines (monocyte chemoattractant proteins and macrophage inflammatory proteins), and growth factors (granulocyte–macrophage colony-stimulating factor and transforming growth factor-β), and has a significant function in aging [13,14]. SASP is responsible for maintaining tissue homeostasis by removing unwanted senescent cells and triggers an inflammatory response that recruits immune cells, such as granulocytes, macrophages, natural killer (NK) cells, and T cells [15]. Thus, if not properly regulated, this system can cause pathological inflammation.

With age, our bodies become more susceptible to harmful inflammatory conditions, such as cytokine storms (a state of excessive cytokine production) [16]. Senescent cells have higher basal levels of various inflammatory cytokines and chemokines [17]. More importantly, in response to IL-1β, lipopolysaccharide (LPS), and tumor necrosis factor (TNF)-α stimulation, the induction of various inflammatory mediators is enhanced in senescent cells compared to that in non-senescent cells, and LPS stimulation causes significantly higher levels of NF-κB nuclear translation [17]. For example, elderly individuals infected with the coronavirus disease 2019 (COVID-19) are likely to have very high rates of adverse health effects and mortality due to cytokine storms [16].

Aging and senescence are caused by various developmental signals and different types of stresses and are considered to be the results of a multistep process [18,19]. Although various induced senescent cell models [20] and aging animal models [2] have been reported to elucidate the complex system, still lacking is an overall picture that recapitulates the cellular transmission and spatial interactions of aging to understand and overcome the unfavorable effects of aging. The utilization of multi-omics methods, such as transcriptomic, epigenetic, and proteomic approaches, has contributed to the elucidation of complex mechanisms. However, these methods cannot determine how age-related diseases can be prevented because they alone cannot generate mechanistic regulatory structures [21]. By contrast, investigating aging and senescence by integrating data-driven time-course omics with mathematical modeling approaches will expand our understanding of how the whole system of aging is regulated and predict the individual outcomes of the system against genetic and environmental factors. Thus, here, ‘systems biology' can be termed as a set of approaches to uncover complex mechanistic structures through analysis of comprehensive time-course omics data and mathematical models to identify, dissect, and manipulate the molecular regulatory network of aging.

In this review, we summarize how NF-κB regulates aging systems and how multi-omics and mathematical modeling approaches have contributed to the elucidation of transcriptional regulation of NF-κB and aging. We also discuss the dynamic properties of the NF-κB system and the significance of oscillation and non-oscillation dynamics in the regulation of downstream target genes.

## NF-κB pathway

Since its discovery as a nuclear factor that binds to DNA elements in the intronic enhancer of the kappa light chain gene of B cells, NF-κB has been investigated for its function as a transcription factor [22]. The NF-κB network consists of seven family members: p105/p50 (*NFKB1*), p100/p52 (*NFKB2*), p65 (*RELA*), RelB (*RELB*), and c-Rel (*REL*); these form homodimers or heterodimers and acquire the ability to bind to DNA differently. Among these proteins, only p65 (*RELA*), RelB, and c-Rel contain the carboxy-terminal transactivation domain (TAD) that activates the transcription of NF-κB target genes [23,24]. In most cells, the p50/p65 heterodimer is a major NF-κB transcription factor. Homo- or heterodimers of p50 and p52 cannot promote transcription due to the absence of TAD but instead bind to the κB site sequence and act as transcriptional repressors [25].

The NF-κB transcription factor constitutes a homo- or heterodimeric protein that binds to a 5'-GGGRNNNYCC-3’ (where G, C, R, Y, and N are guanine, cytosine, purine, pyrimidine, and any nucleotide base, respectively) consensus κB site sequence consisting of 10 nucleotides [26]. The DNA-binding motifs of most transcription factors, including NF-κB, have been identified by the Encyclopedia of DNA Elements (ENCODE) Project Consortium using both chromatin immunoprecipitation (ChIP) and next-generation sequencing [27–29]. To date, several different kB sites have been identified despite being limited to the p50/p65 heterodimer [30].

**Figure 1.**
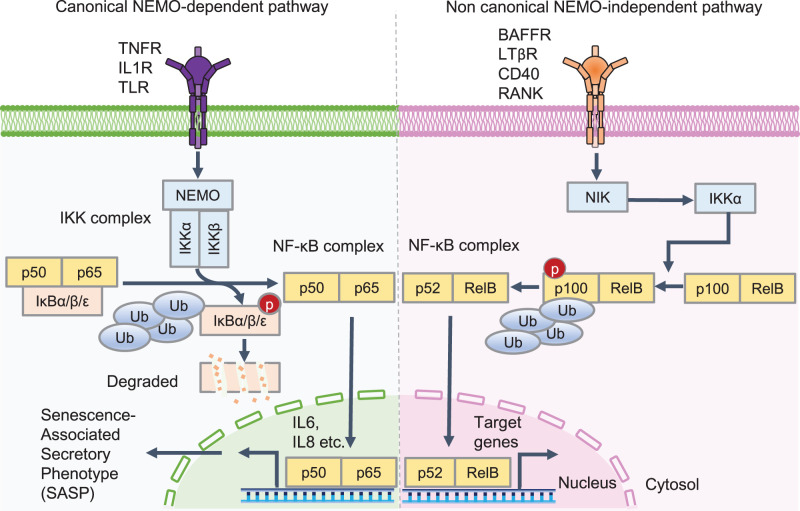
Overview of the canonical and non-canonical NF-κB pathway. In the canonical nuclear factor-κB (NF-κB) essential modulator (NEMO)-dependent pathway, the inhibitor of κB (IκB) kinase (IKK) complex is activated by activated tumor necrosis factor receptor (TNFR), interleukin (IL)-1 receptor (IL1R), and Toll-like receptor (TLR)s. IKK complex activation induces proteasome-mediated proteolysis of IκB proteins and allows the NF-κB complex (p50/p65) to accumulate in the nucleus. p50/p65 dimers bind to DNA and regulate the transcription of senescence-associated secretory phenotype genes, such as IL-6 and IL-8. In the non-canonical NEMO-independent pathway, NF-κB-inducing kinase (NIK) phosphorylates IKKα and leads to the phosphorylation of p100. This process induces subsequent ubiquitination and partial degradation of p100 by the proteasome to form the NF-κB complex (p52/RelB). p52/RelB dimers enter the nucleus and regulate downstream target genes.

The NF-κB network is explained by two major pathways: the canonical NF-κB essential modulator (NEMO)-dependent pathway and the non-canonical NEMO-independent pathway (Figure 1) [31,32]. The canonical pathway is activated by stimuli such as TNF-α, IL-1β, and LPS, which leads to the phosphorylation of the inhibitor of κB (IκB) kinase (IKK) complex; the latter is composed of IKKα, IKKβ, and NEMO. The activation of the IKK complex phosphorylates IκB proteins (IκBα, IκBβ, and IκBε), leading to proteasome-mediated proteolysis of IκB proteins and allowing the NF-κB complex (p50/p65) to enter the nucleus [33].

The non-canonical pathway is NEMO-independent but NF-κB-inducing kinase (NIK)- and IKKα-dependent [34,35]. It is activated through receptors such as B-cell activating factor receptor [36,37], lymphotoxin beta receptor [38], cluster of differentiation (CD) 40 [39], and receptor activator of NF-κB [40]. Signal transduction by receptors activates the NIK/IKKα complex, which phosphorylates p100 and is processed into p52, which allows RelB to form the NF-κB complex (p52/RelB) that moves into the nucleus [34,35]. This translocated complex then binds to DNA and induces its target gene expression, including inflammatory cytokines and modulators of the NF-κB pathway.

## NF-κB and cellular senescence

Accumulating senescent cells is one of the major causes of aging-related disorders [4–6]. It has been reported that senescent cells increase with age in many mice and human tissues, such as the adipose tissue, liver, kidney, and skeletal muscle [41]. Cellular senescence was first reported in 1961 by Leonard Hayflick using human fibroblast cells to relay the limitations of mitotic capacity with replicative stress (RS), known as the Hayflick limitation [42]. Senescent cells are stable, terminal-growth-arrest cells that can be characterized by flat, large-shaped cell morphology, telomere shortening, promotion of cyclin-dependent kinase inhibitor p16 [43] or p21 [44], and increased senescence-associated β-galactosidase (SA-β-gal) activity. SA-β-gal was one of the first published biomarkers of aging, and helped to demonstrate the accumulation of cells with aging characteristics in various mammalian aging-related diseases and in aging tissues [44]. The removal of p16-expressing senescent cells suppresses aging and extends the lifespan of mice, indicating that cellular senescence is an important factor in aging research [43,45].

**Figure 2.**
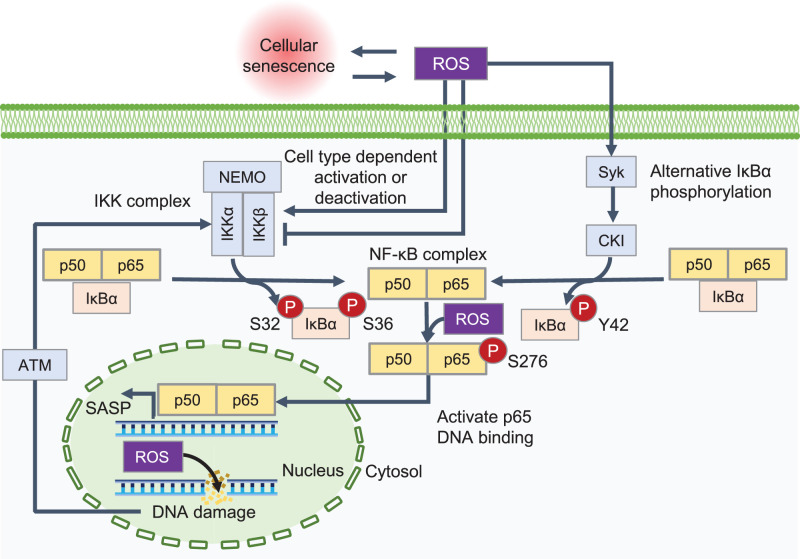
Activation of NF-κB in cellular senescence via ROS. Cellular senescence leads to the accumulation of reactive oxygen species (ROS), which can induce senescence. ROS interact with nuclear factor-κB (NF-κB) at various locations during signal transduction and induce DNA damage. DNA damage induces activation of NF-κB via ataxia-telangiectasia mutated (ATM) kinase activating the inhibitor of κB (IκB) kinase (IKK) complex. ROS are thought to directly affect downstream targets by activating and deactivating the IKK complex, which occurs in a cell type-specific manner. Compared with canonical IKK activation, ROS can alternatively activate the NF-κB complex through the spleen tyrosine kinase (Syk)–casein kinase II (CKII) pathway by phosphorylation of IκBα at Y42. Phosphorylation of p65 at S276 by ROS activates the DNA binding of p65, resulting in greater NF-κB activation (adapted from [47]).

Reactive oxygen species (ROS), such as hydrogen peroxide (H_2_O_2_), and DNA damage accumulate with cellular senescence and crosstalk with NF-κB signaling (Figure 2) [46,47]. ROS induce DNA damage, which results in IKK activation via activation of ataxia-telangiectasia mutated kinase [48]. Typical IκBα is phosphorylated on S32 and S36 by the IKK complex upon NF-κB activation, whereas H_2_O_2_ phosphorylates IκBα on Y42, a process mediated by the spleen tyrosine kinase–casein kinase II pathway [49–51]. H_2_O_2_ directly activates [52–54] or inactivates [55–57] NF-κB via the IKK complex depending on the cell type and influences DNA binding through the phosphorylation of p65 at S276 [58].

In human skin keratinocytes, the overexpression of c-Rel, a subunit of NF-κB, induces premature senescence [59]. The inhibition of H_2_O_2_ in c-Rel-overexpressing keratinocytes decreases the expression of SA-β-gal, suggesting that NF-κB is involved in cellular senescence. In addition, the overexpression of p50/p65 in keratinocytes induces cell growth arrest along with p21 activation [60]. Inflammatory SASPs (such as IL-6 and IL-8, among others) are regulated by NF-κB and not only are characterized as senescence markers but also form autocrine loops and induce growth arrest [61,62]. Microarray analysis of senescent human fibroblasts has shown that the expression of SASP genes is up-regulated and that the inhibition of NF-κB leads to cells escaping cell-cycle arrest [7].

NF-κB maintains cellular senescence by promoting DNA repair and genome stability. Wang et al. [63] compared the rate of immortalization of primary fibroblasts from RelA/p65 (−/−) mouse fibroblasts and RelA/p65 (+/+) cells and found that RelA/p65 (−/−) cells undergo immortalization at a faster rate owing to early DNA mutation and gene deletion caused by genome instability. In a study of NF-κB-dependent effects and mechanisms underlying ROS generation in normal human lung fibroblasts, ROS were reported to cause cell cycle arrest and lead to premature senescence [64]. Interestingly, a previous report showed that the inhibition of NF-κB signaling up-regulates p53-p21 expression and promotes premature senescence [64]. These reports indicate that NF-κB signaling may not only function as a mechanism to promote aging but also have completely opposite functions, suggesting the possibility of differences in functions among cell types and regulatory mechanisms that have not yet been elucidated.

## NF-κB and aging

The role of the NF-κB pathway has attracted increasing attention in the field of aging research [65,66]. Persistent NF-κB activation in chronic inflammation leads to the promotion of aging via the shortening of telomeres [67] and increased expression of γ-H2AX protein [68], which are both widely known aging markers [4,69].

Progressive decline in immune response, including a reduction in proliferative effector responses and a decrease in T cell signaling, has been observed in aging [70]. T cells have several subtypes, including CD8+ T cells (killer T cells), CD4+ T cells (helper T cells), and regulatory CD4+ CD25+ T cells [71]. Human CD8+ T cells are further classified as naïve, central memory (T(CM)), and effector memory cells (T(EM) and T(EMRA)) [72,73]. A Naïve CD8+ T cells and T(CM) CD8+ T cells in the elderly have been found to be more sensitive to apoptosis than those in young individuals [70]. In contrast, T(EM) and T(EMRA) CD8+ T cells are less sensitive, and there is no significant difference in their levels between younger and older individuals [70]. TNF-α production is known to accelerate aging in humans [74–77]. However, the expression of TNF receptors (TNFRI and TNFRII) in CD8+ T cells has been reported to be similar between young and aged subjects [70]. Thus, heterogeneity in the age and cell type response in CD8+ T cells can be attributed to the difference in TNFR downstream NF-κB signal transduction. Because T cells play a central role in cellular immunity, the mechanistic association between NF-κB and aging in T cells will provide a deeper understanding of age-related immune responses.

In terms of the immune response in aging, interesting findings from an animal model of aging, the naked mole-rat (NMR, *Heterocephalus glaber*), have been reported. NMRs are relatively long-lived rodents for their size, and the risk of mortality does not appear to increase with age [78]. Compared with those in mice, macrophages from NMRs have been reported to activate Toll-like receptors in response to LPS stimulation, initiate NF-κB, and produce large amounts of cytokines to enhance immune responses [79]. Hilton et al. [80] collected single-cell RNA sequencing (scRNA-seq) data from spleen derived from NMRs and mice and found NMRs lack canonical NK cells. In addition, LPS was intraperitoneally administered to NMRs and mice, and their spleens were harvested for bulk RNA-seq and scRNA-seq [80]. Subsequently, gene set enrichment analysis (GSEA) revealed that LPS induces the NF-κB inflammatory pathway in both organisms and that NMRs have a subset of LPS-responsive cells that are not present in mice. While no direct reports indicate that NMR aging is linked to NF-κB, elucidation of NF-κB system regulation in NMR may lead to a deeper understanding of aging.

Studies on human fibroblasts from elderly people [11] and animal organs, such as the liver, kidney, and brain, have revealed that the nuclear concentration of NF-κB increases with age. In aging livers, a significant age-related increase in NF-κB binding activity, along with increased levels of nuclear p52 and p65 proteins in the rat liver, has been reported [81]. Interestingly, the expression of NF-κB mRNA (*NFKB1*, *NFKB2*, *RELA*, and *REL*) and its inhibitor transcripts (*NFKBIA* and *NFKBIB*) have not shown statistically significant age-related changes [81]. This result indicates that post-translational modifications occur during liver aging and may affect nuclear localization and binding activity during aging. In rat kidney, decreased IκBα levels and increased nuclear p65 protein levels indicate that NF-κB activity is enhanced by a reduction in IκBα levels [82]. This increase in NF-κB activity is accompanied by an increase in the mRNA and protein levels of cyclooxygenase 2, from which ROS is derived in the body. The roles of IKK-β and NF-κB in mouse brain aging have also been reported. Zhang et al. [10] studied the hypothalamus and cortical tissues of young (3–4-month-old), middle-aged (11–13-month-old), and old (22–24-month-old) C57BL/6 mice and found that p-RelA expression increases in the hypothalamus and cortical tissues as mice age and that preventing the activation of IKK-β and NF-κB in these tissues can prolong their lifespan.

The relationship between NF-κB activation and aging has been demonstrated using aging animal models. Senescence-accelerated mice (SAM), which have been used as an animal model for pathological aging, show increased levels of oxidative stress-related dysfunction and inflammation [83]. SAM prone 8 (SAMP8), a strain of SAM, is short-lived with accelerated aging and exhibits age-related pathologies, such as learning and memory deficits, similar to those in human aging [84]. Pro-inflammatory, pro-apoptotic, and pro-oxidative states are markedly increased in the lungs of aged SAMP8 compared with those of SAM resistant 1 [85]. Forman et al. [85] showed that, in SAMP8 hearts, the nuclear concentration of NF-κB (p50, p52, and p65) is increased, while the levels of cytoplasmic IκBα and IκBβ are reduced.

Experiments on transgenic *Nfkb1*^−/−^ mice have also contributed to uncovering the relationship between NF-κB and aging [2]. *Nfkb1^−/−^* mice lack both the p105 precursor and p50 subunits and demonstrate persistent induction of p65/p65 homodimer formation [86]. The phenotype of this transgenic mouse shows accelerated apparent senescence, shortened lifespan, telomere damage, and a chronic inflammatory state, which leads to the production of SASP. A cellular level analysis of *Nfkb1^−/−^* mice showed decreases in proliferation speed and apoptosis and increases in SA-β-gal positive cells, expression of cyclin-dependent kinase inhibitors p16 and p21, and γ-H2AX accumulation [87]. These data indicate that the loss of *Nfkb1* leads to premature senescence in mammals.

## Multi-omics regulation of NF-κB and aging

What occurs within our bodies as we age? To answer this question, multi-omics analysis has been vigorously performed to understand transcriptomic, epigenetic, and proteomic regulation [88]. Attempts to collect omics data on aging phenotypes have produced several databases, such as Human Aging Genomic Resources (HAGR) [89], SeneQuest [90], and the Aging Atlas [91], which help us to integrate data and provide a bird's eye view of the biological pathways involved in the aging process. HAGR (http://genomics.senescence.info/) is a collection of databases on human and model organism aging that can be searched for age-related genes, drugs, and genetic variants [89]. SeneQuest (https://senequest.net/) is a database of genes related to senescence and is maintained by the International Cell Senescence Association [90]. The Aging Atlas (https://ngdc.cncb.ac.cn/aging/index) is a multi-omics database for aging biology that enables researchers to find information from genomics, epigenomics, transcriptomics, proteomics, metabolomics, and pharmacogenomics data at either the bulk or single-cell level [91]. These databases were developed with graphical user interface-based platforms and enable biologists to readily access omics data related to aging and senescence.

Hereafter, we discuss omics research investigating the relationship between NF-κB and aging (Table 1).

**Table 1:** Omics studies of NF-κB and aging

Omics	Methodology	Sample	Result	Reference
Transcriptome regulation	Microarray	Naïve (CD44^low^) and memory (CD44^high^) CD4+ T cells derived from young (2–3-month-old) and aged (28-month-old) mice	Upstream analysis of differentially expressed genes during aging was performed, and NF-κB was identified as a potential age regulator	[92]
Transcriptome regulation	RNA-seq	pHBEC senescence induced by RS and CSE	Under both RS and CSE stress, gene set enrichment analysis indicated dysfunction in the regulation of ROS, proteasome degradation, and NF-κB signaling	[93]
Transcriptome regulation	miRNA sequencing	Peripheral arterial and venous blood of young (8-week-old) and aged (22-month-old) rats	miR-136-3p and miR-503-3p are differentially expressed with aging and are regulated by NF-κB and SIRT1	[95]
Transcriptome regulation	scRNA-seq	Lung, heart, and artery tissues derived from young (4–6-year-old) and old (18–21-year-old) cynomolgus monkeys	ACE2 expression increases with age in alveolar epithelial barrier, cardiomyocytes, and vascular endothelial cells; IL-7 accumulates in aged cardiopulmonary tissues and induces ACE2 expression in human vascular endothelial cells in an NF-κB-dependent manner	[98]
Transcriptome regulation	Microarray	Sun-unexposed skin tissue of healthy males aged 19 to 86 years	Metabolic activity and cellular damage associated with NF-κB pathways increase in the middle aged (30–45 years old)	[99]
Transcriptome regulation	scRNA-seq	Human upper eyelid skin samples collected from young (18–28-year-old), middle-aged (35–48-year-old), and aged (70–76-year-old) groups	NF-κB signaling pathway is up-regulated with aging in several cell types, including epidermal basal cells, mitotic cells, granular cells, and spinous cells	[101]
Transcriptome and epigenetic regulation	RNA-seq ChIP-seq (H3K27ac)	Brain tissues derived from young (<60-year-old) and old (>60-year-old) humans and young (3-month-old) and aged (18-month-old) mice	The expression levels of regulators of the NF-κB pathway, *TNFRSF1A*, *NFKBIA,* and *TMED4*, increase in the aged brain, and NF-κB and BCL3 binding annotations are concentrated in up-regulated genes	[106]
Transcriptome and epigenetic regulation	Microarray ChIP-seq	ChIP-seq data from ENCODE [27–29] and the list of kidney age-regulated genes from Rodwell et al. [12]. Additionally, TNF-, IFNγ-, and IL-6-treated human renal proximal tubular epithelial cells	Both transcriptomic and epigenetic analyses showed that the expression levels of NF-κB, STAT1, and STAT3 increase with renal aging	[108]
Transcriptome and epigenetic regulation	RNA-seq ChIP-seq (H3K4me3 and H3K27ac)	Heart, liver, cerebellum, and olfactory bulb derived along with primary cultures of neural stem cells from young (3-month-old), middle-aged (12-month-old), and aged (29-month-old) mice	Both transcriptomic and epigenetic analyses of heart, liver, and cerebellum and functional enrichment analysis showed TNF-α signaling via NF-κB is up-regulated as age increases	[109]
Protein regulation	Nano LC-MS/MS	Senescent human diploid IMR-90 fibroblasts induced by etoposide or infection with oncogenic H-Ras^V12^	In senescent IMR-90 cells, the NF-κB p65 subunit was found to be one of the most significantly enriched transcriptional regulators bound to chromatin	[113]
Protein regulation	Nano LC–MS/MS	Marmoset senescent TPC induced by RS and TPC from young (2–3-year-old) and aged (10–15-year-old) marmoset monkeys	In both the RS *in vitro* model and aged cell *in vivo* model, NF-κB signaling is altered by aging	[114]
Protein regulation	Nano LC–MS/MS	Tear samples from health humans (18–83 years old)	Upstream analysis of 17 tear fluid-derived proteins, which were correlated with donor age, showed that the NF-κB complex acts as a transcriptional regulator	[115]

Abbreviations: NF-κB, nuclear factor-kappa B; pHBEC, primary human bronchial epithelial cell; RS, replicative stress; CSE, cigarette smoke extract; ROS, reactive oxygen species; SIRT1, sirtuin-1; scRNA-seq, single-cell RNA sequencing; ACE2, angiotensin-converting enzyme 2; ENCODE, Encyclopedia of DNA Elements; TNF, tumor necrosis factor; IFN, interferon; IL, interleukin; STAT, signal transducer and activator of transcription; Nano LC–MS/MS, nanoscale liquid chromatography coupled to tandem mass spectrometry; TPC, testicular peritubular cells.

## Omics 1. Transcriptome regulation of NF-κB and aging

Transcriptomic changes during the aging of various cells and tissue samples indicate a relationship between NF-κB and aging. To explore the cause of the decline in immune response, transcriptomic profiles of naïve (CD44^low^) and memory (CD44^high^) CD4+ T cells, which are impaired by the aging process, were derived from young and aged mice and examined by Taylor et al. [92]. To determine probable upstream regulators, cis-regulatory analysis of differentially expressed genes during aging was performed, and NF-κB was identified as a potential age regulator [92].

Transcriptome changes in senescent cells induced by RS and cigarette smoke exposure also indicate that NF-κB is related to aging [93]. Cigarette smoke exposure and RS are well-known senescence inducers [94]. Voic et al. [93] performed RNA-seq in passaged cigarette smoke extract (CSE)-exposed and non-CSE-exposed senescent primary human bronchial epithelial cells and showed that GSEA indicated dysfunction in the regulation of ROS, proteasome degradation, and NF-κB signaling under either RS or CSE. miRNA analysis has also been conducted to explore the influence of aging. Transcriptome analysis of miRNA from the peripheral blood of young and aged rats showed that miR-136-3p and miR-503-3p are differentially expressed with aging and are regulated by NF-κB and SIRT1 [95].

Aging has been reported to increase the risk of severe COVID-19 [96,97]. Ma et al. [98] created a single-cell transcriptomic atlas of the cardiopulmonary system of aged cynomolgus monkeys and reported the involvement of NF-κB in age-related susceptibility to severe acute respiratory syndrome coronavirus disease 2 (SARS-CoV-2). The authors reported that the expression of angiotensin-converting enzyme 2 (ACE2), a receptor for SARS-CoV-2, increases with age in the alveolar epithelial barrier, cardiomyocytes, and vascular endothelial cells. They also found that aged cardiopulmonary tissues accumulate IL-7 and induce ACE2 expression in human vascular endothelial cells in an NF-κB-dependent manner.

Interestingly, the aging of the skin is thought to differ slightly from that of other tissues because it is caused by both internal factors (such as senescent cells) and external factors (such as ultraviolet light [UV]). Haustead et al. [99] focused on skin aging caused by internal factors by analyzing the transcriptome of sun-unexposed skin tissue from healthy males aged 19 to 86 years. In middle-aged subjects (30–45 years old), the authors found gene enrichment in response to DNA damage stimuli and positive regulation of the NF-κB cascade via IKK. Regarding the relationship between UV light and NF-κB, O'Dea et al. [100] reported that UV light does not induce but amplifies NF-κB activity. Using mathematical modeling, the authors showed that UV-induced activation of NF-κB occurs not only through the IKK-mediated degradation pathway of IκBs but also through the IKK-independent pathway. Single-cell transcriptomic analysis has also been used to reveal the involvement of NF-κB in human skin aging. Zou et al. [101] collected scRNA-seq data from the eyelid skin of healthy individuals of various age groups and identified 11 canonical cell types and 6 basal cell types in skin tissues. Their analysis showed that the NF-κB signaling pathway was up-regulated with aging in several cell types, including epidermal basal cells, mitotic cells, granular cells, and spinous cells.

## Omics 2. Epigenetic regulation of NF-κB and aging

When histones are subjected to epigenetic modifications, such as methylation and acetylation, chromatin changes its shape to form either euchromatin, where transcription factors can likely bind to DNA and transcription is actively carried out, or heterochromatin, where DNA aggregates and transcription factors cannot easily bind, which can be monitored by ChIP-seq [102,103]. Epigenetic changes have been shown to be a feature of aging [69]. For example, disturbance of histone H3 trimethylation at lysine 4 or lysine 27 (H3K4me3 or H3K27me3, respectively) has been reported to affect the lifespan of *Caenorhabditis elegans* [104,105]. In the brains of aged humans and mice, a general loss of H3K27ac has been observed [106]. In addition, deletion of SIRT6, a deacetylase of H3K9ac, results in excessive activation of NF-κB signaling and accelerated aging [107]. Here, we introduce epigenetic studies that elucidate the relationship between NF-κB and aging.

Brown et al. compared transcription factor changes during kidney aging using data from the ChIP-seq dataset from the ENCODE Consortium [27–29] and genome-wide maps of transcription factor occupancy obtained from ChIP-seq data of human cells [108]. The authors identified associations between ENCODE data and kidney age-related genes derived from transcriptional profiles [12] and found NF-κB, signal transducer and activator of transcription (STAT)1, and STAT3 to be promising candidates whose activities increase with age in the epithelial compartment of the renal cortex [108]. They also showed that DNA variants common to *RELA* and *NFKB1* are associated with renal function and chronic kidney disease in genetic association studies, showing that genetic variants in NF-κB contribute to the phenotype of renal aging.

Chronic inflammation aging profiling using RNA-seq and H3K27ac ChIP-seq of human and mouse brain samples was performed by Cheng et al. [106]. The expression levels of regulators of the NF-κB pathway — *TNFRSF1A*, *NFKBIA*, and *TMED4* — were increased in the aged brain; NF-κB and BCL3 binding annotations were enriched in up-regulated genes, whereas the expression of GATA-3, which is related to the regulation of neuronal functions, was down-regulated [106].

Systematic studies of transcriptomic and epigenomic changes in several tissues and species during aging have been reported. Benayoun et al. [109] generated ChIP-seq (H3K4me3 and H3K27ac) and RNA-seq datasets from tissues of young, middle-aged, and old C57BL/6N male mice. Transcriptomic, epigenomic analyses of the heart, liver, and cerebellum and functional enrichment analysis showed that TNF-α signaling via NF-κB increases as age increases [109]. The authors also used public human [110], rat [111], and African turquoise killifish (*Nothobranchius furzeri*) [112] transcriptome data to determine whether the effects of aging are conserved in multiple vertebrates; they found that functional enrichment showed TNF-α signaling via NF-κB is commonly regulated by aging.

## Omics 3. Protein regulation of NF-κB and aging

While genetic and epigenetic regulation is important, it is also essential to understand the function of proteins to identify changes in physiological phenomena. Here, we introduce the relationship between NF-κB and aging at the proteomic level. Chien et al. [113] performed a proteomic analysis of proteins bound to chromatin in senescent IMR-90 cells and found that the NF-κB p65 subunit is one of the most significantly enriched transcriptional regulators. Stöckl et al. [114] reported proteome and secretome analyses of RS induced in testicular peritubular cells derived from marmoset monkeys and showed impaired protein secretion, altered NF-κB signaling, and reduced contractility.

The effect of aging on tear fluid samples and its association with NF-κB was reported by Nättinen et al. [115], who performed proteomics analysis using healthy human tear samples collected from 115 subjects. In their study, 17 tear proteins related to inflammation, immune response, and cell death were found to correlate with donor age. Upstream analysis of these proteins indicated that the NF-κB complex acts as a transcription regulator.

*In vitro* and *in vivo* multilayer omics analyses have shown that NF-κB signaling is highly related to senescence and aging. Data-driven omics analyses in aging have revealed that the perturbation of aging activates the promoter and enhancer regions of NF-κB target genes, promotes binding of NF-κB to the κB site, and increases transcription of NF-κB target genes.

## Mathematical modeling of NF-κB and aging

**Figure 3.**
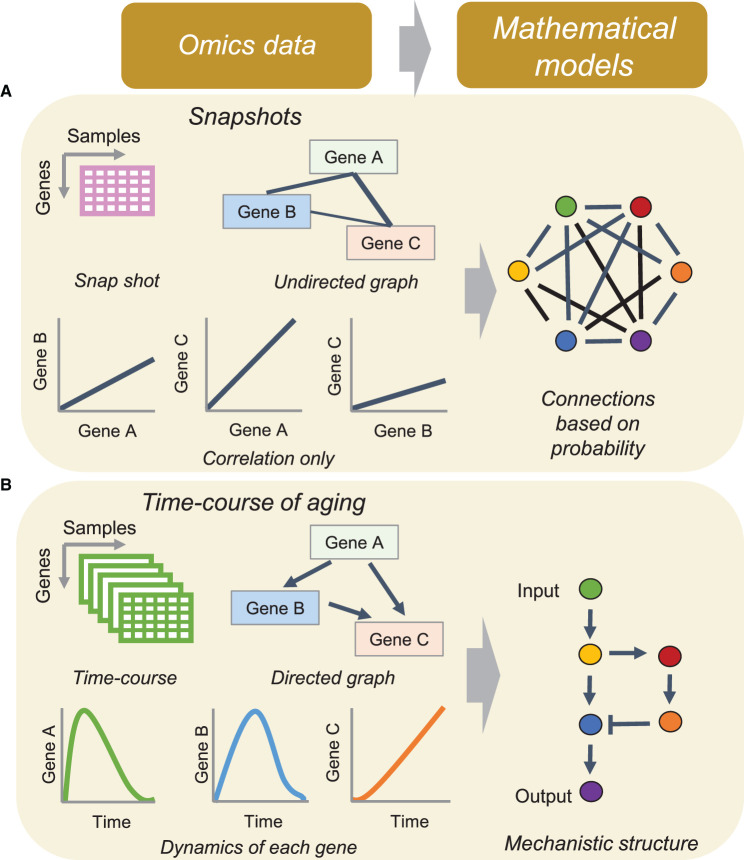
Differences between time-course and snapshot omics analysis. (**A**) Time-course aging omics showing a directed graph between the gene regulatory network and the dynamics of each gene. By integrating these data with mathematical models, mechanisms of biological functions can be obtained. (**B**) Snapshot omics studies do not provide information on the underlying regulatory mechanisms but provide only information regarding the correlations between genes. For these datasets, we can only construct mathematical models based on the probability of correlation among genes.

Data-driven omics analysis provides valuable information regarding the relationship between NF-κB and aging. Snapshot omics studies do not provide information on the underlying mechanistic regulatory structures; only the correlations between genes based on their expressions can be obtained [21]. From these static datasets, we can construct only mathematical models (e.g. regression models [116]) based on the probability of correlation between genes (Figure 3A). By contrast, time-course omics data on aging, along with knowledge of biological aging, show a directed graph between the gene regulatory network (GRN) and the possible activity dynamics of each gene. By integrating these data with mathematical models and performing numerical simulations, we can predict the mechanistic structure of biological systems and even manipulate them (Figure 3B). Thus, integration between time-course omics data and mathematical models can provide a mechanistic structure to understand the entire system of aging.

Dynamic behaviors obtained from a mathematical simulation can also provide important insights into biological functions *in silico*, elucidating sensitivity and robustness of regulatory mechanisms and filling the gap between experimentally observable data and theoretical regulatory principles [117,118]. Biological systems can be represented using several networks, such as the ordinary differential equation (ODE) model, Boolean model, and Bayesian network [119–121].

ODE models are most commonly used to quantitatively understand biological systems [122,123]. The ODE model is said to be ordinary because it contains only one independent variable, which is basically time. It assumes that species are present in well-mixed compartments and that concentrations can be considered to be continuous [119], which generally makes the ODE model unsuitable for representing processes such as diffusion, spatial heterogeneity, and stochasticity [124]. Each variable represents the concentration of one component (e.g. gene), and how it changes over time depends on the initial value of each variable, the concentrations of other variables, and fixed kinetic parameters. Hence, if we attempt to represent the biological system with the ODE model, we need to estimate the kinetic parameters that correspond to the time-series data, which increases the computational cost by expanding the model.

The Boolean network is a type of dynamic model that is based on quantitative logical rules [120,121,125]. Based on the interaction between genes, regulation (e.g. transcriptional regulation) can be termed as active or inactive. In other words, this can be stated as Boolean logical values: OFF (‘0') or ON (‘1'). The regulation network between nodes, for example, genes, phosphorylation, or ubiquitination, can be connected with ‘logic gates' found in digital electronic circuits (e.g. AND, OR, and NOT gates) [120,121]. Each logic gate has a set of logical rules that connect the input to the output. Time in the Boolean network takes integer values, and nodes are either ‘OFF' or ‘ON' depending on the activation or repression, respectively, of each factor, which allows us to simulate dynamic behavior.

A Bayesian network reconstructs a gene regulatory expression network between nodes to statistically evaluate different connections in a network with conditional probability [126,127]. One characteristic of the Bayesian network is that it simplifies high-dimensional data into a simple graph. In a Bayesian network graph, the nodes represent the variables (e.g. gene), and the edges represent the links between genes. Displaying probability distributions as a directed graph makes graphical analysis possible. Calculating these probabilities according to the occurrence path makes it possible to quantify the probability of the occurrence of causal relations with complex pathways.

Mathematical models using the Boolean network have been proposed to elucidate the regulatory network between NF-κB and aging [128–130]. A Boolean network model-based GRN of the SASP activated by DNA damage was proposed by Meyer et al. [128]. They simulated a model of pathway interactions between p53/p16-induced cell cycle arrest, NF-κB-regulated SASP, and IL-1/IL-6-driven inflammatory activity based on knowledge of biological aging. From their work, NEMO was identified as a target for mechanistic inhibition of IL-6 and IL-8 and was experimentally validated using NEMO knockout murine dermal fibroblasts.

In addition to the knowledge-based mathematical model, Schwab et al. integrated data-driven omics analysis and the Boolean model to clarify hidden knowledge under NF-κB signaling and aging [129,130]. The authors used a binarized time-series gene expression dataset of muscle samples [131] from young and elderly healthy male humans to construct a Boolean network and found behavioral changes in NF-κB signaling during aging [129].

A combination of sc-RNA-seq of human hematopoietic stem cells (HSCs) derived from young (19–40-year-old) and aged (61–70-year-old) human individuals [132] and a Boolean network was used to capture the heterogeneity of aging in NF-κB signaling-regulated genes [130]. With aging, the number of HSCs increases, but their activity becomes highly heterogeneous and impaired, which is known to be related to NF-κB signaling-induced inflammation [133]. Using pseudo time-series of single-cell data in dormant and active HSCs, Schwab et al. [130] reconstructed specific regulatory networks for each individual and performed attractor analysis to show that single-cell dynamics of NF-κB target genes capture heterogeneity in mechanisms and regulatory networks of aging HSCs. This study shows that the integration of omics analysis and mathematical models is a powerful tool to elucidate hidden mechanisms between NF-κB and aging. Moreover, it might be possible to elucidate transcriptional heterogeneity in other tissues using scRNA-seq aging data with time-course omics and mathematical integration methods [134].

Notably, aging is a physiological phenomenon that progresses over several decades. By contrast, cellular senescence can be induced by a variety of stimuli in a rather short time period [20]. In other words, time-series information on signal transduction is important for phenomena that change rapidly, such as cellular senescence, and a model that allows a structural understanding of signal pathways, such as the ODE model, is considered suitable. Thus, the selection of an appropriate model for each dataset in aging and senescence should be considered before building the model.

## Dynamic properties of NF-κB regulation

Based on scRNA-seq analysis of aging cells and tissues, such as T-cells [135], aorta and coronary arteries [136], pancreas [137], microglia cells [138], lung [139], skin [101,140], and muscle [141], transcriptional heterogeneity has been reported to increase with aging [134]. Understanding the heterogeneity in aging will make it possible to identify new markers that distinguish between actual and biological age and provide optimal treatment options for individuals [142]. Moreover, single-cell dynamics of NF-κB target genes capture heterogeneity in the mechanisms and regulatory networks of aging HSCs [130]. Dysregulation of nuclear localization of NF-κB leads to various pathological conditions [143]; therefore, controlling the localization of NF-κB might shed light on the heterogeneity in aging, and research on the regulation of NF-κB dynamics is needed. Thus, in this chapter, we first introduce what has been elucidated in the regulatory systems of NF-κB dynamics, then examine how NF-κB dynamics change the response to different types of input stimuli from mathematical research findings.

**Figure 4.**
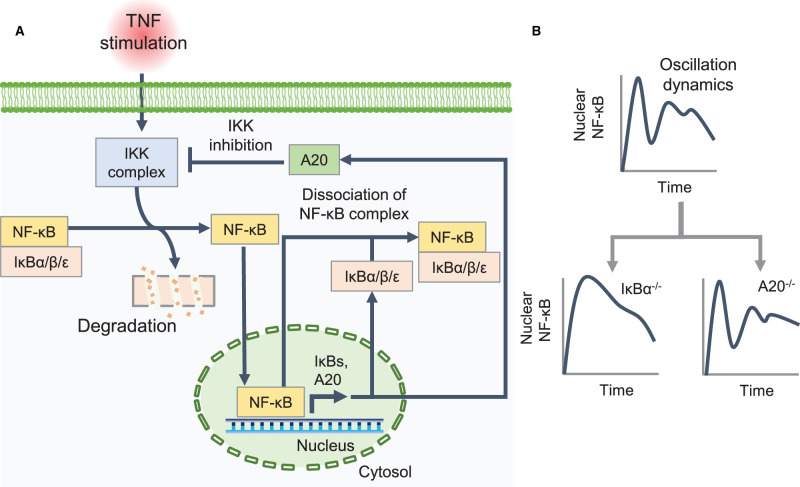
Negative feedback mechanism in NF-κB dynamics. (**A**) Upon inhibitor of κB (IκB) kinase (IKK) complex activation by tumor necrosis factor (TNF) stimulation, nuclear factor-κB (NF-κB) is released by IκBs (IκBα, IκBβ, and IκBε) and enters the nucleus. Along with the activation of inflammation-related genes, NF-κB induces IκBs and A20. When NF-κB is activated, IκBs are released from the NF-κB DNA-binding domain, and A20 suppresses IKK activation. IκBα, which rapidly transfers to the nucleus upon synthesis, is the primary component of this negative feedback and has the function of stripping NF-κB bound to DNA and transferring it to the cytoplasm. Due to this negative feedback system, NF-κB moves in and out of the nucleus, resulting in an oscillating behavior. (**B**) *IKBA* knockout abolishes NF-κB oscillations. By contrast, A20 mainly shapes the late NF-κB response rather than the initial response, and thus, knockout of *TNFAIP3* does not directly affect NF-κB localization (adapted from [147]).

Along with the activation of inflammation-related genes, NF-κB has multiple negative feedback mechanisms and constitutes a complex regulatory mechanism. These negative feedback loops shape the characteristic oscillation dynamics of NF-κB nuclear localization (Figure 4). IκBs (*NFKBIA* [IκBα], *IKBKB* [IκBβ], and *IKBKE* [IκBε]) and *TNFAIP3* (A20) are target genes of NF-κB; when NF-κB is activated, IκBs are released from the DNA binding domain of NF-κB, and A20 suppresses IKK activation [144–146]. These two negative feedback processes have distinct functions in the regulatory system of NF-κB dynamics. IκBα protein is synthesized upon stimulation of the NF-κB pathway and rapidly transferred to the nucleus; it strips NF-κB bound to DNA and transfers it to the cytoplasm [145]. Therefore, knockout of *IKBA* abolishes NF-κB oscillation, which induces sustained activation of NF-κB target genes [147,148]. IκBβ, which has high homology with IκBα, is reported to be unable to provide the same adequate negative feedback as IκBα [149]. In contrast to IκBα, IκBε has been reported to show delayed negative feedback due to transcriptional delay [150]. Using the ODE model, Longo et al. [151] showed that this delayed negative feedback by IκBε controls dampened oscillation of NF-κB. Interestingly, A20 mainly shapes the late NF-κB response rather than the initial response, and thus, knockout of *TNFAIP3* does not directly affect NF-κB localization [147].

Many mathematical models include a negative feedback mechanism by IκBs or A20 in NF-κB signaling to identify the mechanism of NF-κB oscillations or their encoding or decoding properties. The difference between oscillating and non-oscillating NF-κB dynamics was investigated by Mothes et al. [152] using the ODE model. They found that NF-κB can exhibit heterogeneity of dynamics from the same stimulus, which is caused by a change in intracellular parameters in the pathway. They also indicated that the concentration of NF-κB and the transcriptional rate constant of IκBα are important parameters that change the dynamics and fold change of NF-κB. Benary and Wolf extended the ODE model developed by Lipniacki et al. [153] to investigate the impact of the regulation of beta-transduction repeat containing protein (β-TrCP)-mediated IκB degradation [154]. NF-κB is activated when IκBs are degraded, and this process is mediated by β-TrCP. In this study, they found that enhancing the β-TrCP-mediated degradation of IκB increases the steady-state concentration of nuclear NF-κB.

A20 is also an important feedback system, along with IκBs. Mothes et al. [155] used a modular modeling approach to determine the impact of different A20 feedback implementations on the dynamics of NF-κB. The authors used three different models (Lipniacki et al. [153], Ashall et al. [146], and Murakawa et al. [156]), which had different A20 implementations against IKK inhibition, and combined them with a common IκBα–A20 negative feedback core module. The authors also analyzed the effects of a wide range of changes in A20 feedback strength, IκBα feedback strength, and TNF-α stimulus strength on NF-κB dynamics. The results showed that A20 feedback strength and TNF-α stimulation strength had different effects on the initial and long-term NF-κB concentrations depending on the models analyzed. Based on experimental validation using TNF-α-stimulated HeLa cells, Murakawa et al. [156] best described the dynamic features of NF-κB. This indicates that regulation described in their ODE model [156], in which both TNF-α-dependent and -independent regulation increases the amount of active IKK and its inhibition by A20, is imprinted in TNF-α-stimulated HeLa cell systems. Using their modular modeling approach, we can elucidate the mechanism by which A20 negative feedback inhibits IKK depending on the cell type.

Positive feedback regulates the TNF-α pathway. A positive feedback mechanism within the upstream kinase signaling complex induces the switch-like activation of NF-κB [157]. TNF-α itself is also transcriptionally regulated by NF-κB, which is reported to function as a feedforward-like rather than positive feedback regulation because several additional regulations, such as splicing control, mRNA degradation, pro-TNF-α activation, and secretion, are needed to fully activate TNF-α [144,158].

Recent reports indicate that the oscillation dynamics of NF-κB nuclear localization are functionally important for biological systems [148,159]. Adelaja et al. [159] reported that different stimuli, such as TNF-α, Pam3-Cys-SK4 (P3CSK), polyinosinic:polycytidylic acid (Poly(I:C)), LPS, and CpG, induce the activation of NF-κB with different dynamics. Using the features of different stimuli and machine learning, they identified six dynamic features encoding NF-κB dynamics: activation speed, peak amplitude, oscillatory dynamics, total activity, duration, and ratio of early to late activity. They further utilized the ODE model to visualize the underlying mechanism and indicated four key characteristics: ligand half-life, receptor translocation and replenishment rates, dose response of adaptor proteins, and deactivation kinetics of adaptors. This study illustrated that cytokine stimuli (TNF-α) and bacterial pathogenic stimuli (P3CSK, Poly(I:C), LPS, and CpG) are recognized by cells as differences in dynamics and induce different biological functions.

Cheng et al. [148] identified NF-κB dynamics as important properties regulating epigenetic changes induced by different input stimuli in macrophage activation. In particular, they showed that non-oscillatory NF-κB liberates chromatin by disrupting nucleosome–histone and DNA interactions. This finding not only indicates that NF-κB oscillation influences chromatin accessibility and regulates gene expression but also presents important evidence that NF-κB dynamics have significant implications for biological systems.

scRNA-seq has been used to reveal the heterogeneity of NF-κB dynamics. Lane et al. [160] revealed the relationship between live-cell imaging of nuclear NF-κB dynamics and scRNA-seq gene expression induced by LPS stimulation using the same single cell. By comparing clustered cells based on NF-κB dynamics observed by microfluidic single-cell imaging and scRNA-seq, the authors found that strong and long-lasting nuclear p65 signaling correlated with increased expression of pro-inflammatory cytokines. Since pro-inflammatory cytokines increase with aging, sustained nuclear p65 activation might be a promising target for the treatment of aging.

As shown in this chapter, the nuclear NF-κB localization pattern is regulated by multiple feedback mechanisms, and NF-κB dynamics are meaningful for the regulation of downstream target genes. Mathematical models, in particular the ODE models, have played important roles in elucidating NF-κB dynamics. How nuclear NF-κB dynamics are involved in aging and what the results of NF-κB dynamics disturbance would be in terms of aging remain unclear; however, these areas seem to be promising targets for preventing age-related diseases.

Next, we will look closer at NF-κB mathematical models for a deeper theoretical understanding of NF-κB dynamics and consider the possibility of regulating aging.

## Single-cell analysis and mathematical modeling of the NF-κB network

The unique properties of oscillation dynamics of the NF-κB signaling pathway have been studied and reviewed using mathematical models [161–165]. As previously mentioned, the dynamic properties of NF-κB regulation during aging are not yet fully understood. Thus, utilization of NF-κB mathematical models in aging phenomena, such as decline in immune response, may provide clues to understanding the mechanistic structure of NF-κB dynamics. Here, through a mathematical model of NF-κB, we explore how the heterogeneity of NF-κB dynamics is regulated at the single-cell level and aim to gain insights into the relationship between NF-κB dynamics and aging.

The control of heterogeneity in NF-κB dynamics has been studied along with the development of technologies that allow live imaging observation of single cells; for instance, microfluidic devices allow constant imaging under various conditions specified by the researcher.

When NF-κB activity is measured at the bulk population level using methods such as western blot (WB) analysis, the behavior of single cells is masked in the underlying system [166]. For example, an analog response to a gradual increase in activation and an all-or-nothing digital response may produce similar WBs even though the number of activated cells and the amount of activity per cell may be different [166]. Thus, single-cell level observation is a powerful tool to elucidate NF-κB heterogeneity.

Cells regulate inflammation, caused by cell damage or microbial invasion, by detecting changes in the surrounding environment through NF-κB signaling. However, in the actual cellular environment, the concentration of NF-κB ligands around the cells may fluctuate randomly even when no immune or inflammatory response occurs. Therefore, the NF-κB pathway must distinguish between this external ‘noise' and significant increases in ligands associated with the immune response. To overcome this problem, a system regulated as ‘ON’ or ‘OFF,' called the switch-like system, is activated only when a sufficiently strong signal exceeding the internal threshold is applied in NF-κB signal transduction [167–169]. This increases the robustness of cellular decision-making in noisy environments, resulting in cell-to-cell heterogeneity in transcriptional response [170,171].

To clarify the heterogeneity of NF-κB activation among single cells, Kellogg et al. [167] and Tay et al. [169] focused on the encoding and decoding of the duration and concentration of ligand of the input information by the NF-κB system. Experimental observation of single-cell NF-κB activation by LPS [167] or TNF-α [169] treatment suggested that NF-κB activation at the microscopic level is similar to a switch, with the strength of the induced signal altering the stochastic all-or-nothing response in each cell. Using a hybrid stochastic–deterministic mathematical model, Kellogg et al. [167] found that the integral value (calculated by multiplying ‘concentration' by ‘duration') of stimulation determines the activation of NF-κB in single cells and that the sustained weak stimulation results in heterogeneous activation and timing delay, which is transmitted to gene expression. By contrast, transient strong stimulation at the same site results in rapid and uniform dynamics [167]. These results indicate that it is possible to control the phenotype of a cell via the strength of the input and to control the activation probability of a single cell. They also indicate that a switch-like NF-κB system response to bacterial pathogenic stimuli results in a heterogeneous reaction in the cell population and protects against chronic activation by external ‘noise' in the environment. With aging, SASP and chronic inflammation (inflammaging) occur; the ‘concentration' and ‘duration' of NF-κB perturbation may be said to increase with aging, causing an ‘ON' state in the switch-like NF-κB system.

To understand the sources of internal ‘noise' under intercellular heterogenic responses in the NF-κB system, Bass et al. [172] combined single-transcript measurements with mathematical models to study transcriptional noise in NF-κB-regulated genes. A main cause of gene expression noise in a single cell is the flow of promoters between transcriptionally active and inactive states, a process known to induce ‘transcriptional bursting' [173]. By analyzing the changes in gene expression noise and transcriptional bursts at endogenous NF-κB target promoters before and after TNF-α stimulation, they found that TNF-α primarily activates transcription by increasing the burst size while maintaining burst frequency at gene promoters with relatively high basal values of histone 3-acetylation, which marks the open chromatin status [172]. The authors simulated the transcriptional bursting behavior using a two-promoter state model in which a promoter switches the ‘OFF' state to the ‘ON' state [172]. In this model, the transcription process is described by two main features: burst size, defined as the average number of mRNAs produced per burst (gene activation event), and burst frequency, defined as the frequency at which bursts occur [174]. Using this model, Bass et al. [172] showed that positive feedback of TNF-α amplifies the noise of gene expression by transcription through burst size, forming a subset of cells that express high levels of TNF-α protein. The effect of ‘transcriptional bursting' in aging has not been reported, but since the levels of TNF-α are known to increase with aging [74–77], this internal ‘noise' might provide clues to elucidate the heterogeneity in aging between cells and individuals.

To understand whether cells respond to absolute cytokine levels or to the rate of change in the dynamic concentration of cytokines around the cells, Son et al. [175] studied how the NF-κB pathway responds to time-varying immune inputs, such as increases, decreases, and fluctuations in cytokine signaling. From experimental single-cell observations, they found that NF-κB activity responds to differences in absolute cytokine concentrations, not the cytokine concentrations themselves. Sensitivity analysis using their ODE modeling suggested that A20 and IκBα negative feedback mechanisms accurately detect the dynamics of extracellular cytokines. This work indicates that changes in environmental cytokine concentrations are important for signal processing and that expression of some NF-κB target genes (*TNFAIP3* and *CCL2*) correspond closely to the TNF-α dose at a given time during ramping.

**Figure 5.**
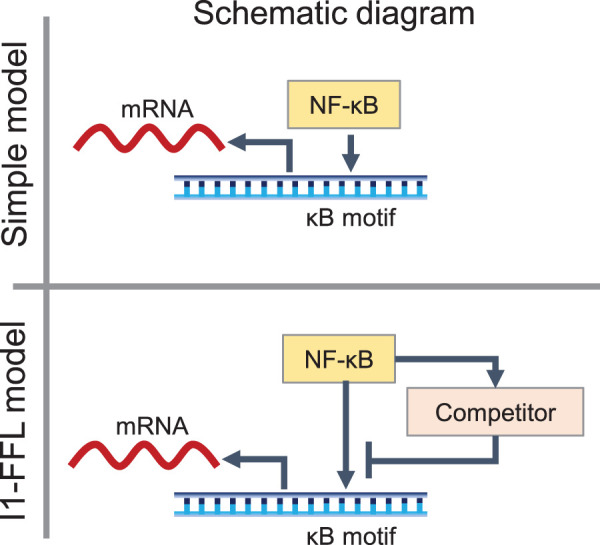
Schematic diagrams of simple and I1-FFL models. In a simple model, nuclear factor kappa B (NF-κB) binds to the κB motif, which results in the transcription of downstream target genes. In the incoherent feedforward loop (I1-FFL model), NF-κB and its competitors competitively bind to the κB motif and initiate transcription of downstream target genes.

The impact on the rate of change in the localization of NF-κB has also been reported [176]. Nuclear NF-κB levels vary considerably among cells, even in unstimulated cells. To elucidate the mechanism underlying this heterogeneity, Lee et al. [176] confirmed the localization of p65 (*RELA*) upon TNF-α stimulation in single cells, indicating that the rate of change rather than the absolute amount present in the nucleus is important for the transcriptional activity of NF-κB. Using mathematical modeling, Goentoro et al. [177] found that an incoherent feedforward loop (I1-FFL) (Figure 5) initiated by competition for κB motif binding provides the pre-ligand state required for fold-change detection [176]. In the I1-FFL model, NF-κB and its competitors competitively bind to the κB motif and transcribe the target genes downstream. In the absence of I1-FFL regulation, the transcriptional dynamics of *IL-8*, *TNFAIP3*, and *NFKBIA* could not be reproduced when TNF-α stimulation was applied to experimentally obtained dynamics, whereas the I1-FFL model reproduced the experimental dynamics [176]. The I1-FFL model involves transcription and translation of the competitive factor, and its inhibition results in a structure that causes a time delay [176]. These findings indicate that the transcriptional activity of NF-κB is controlled by two contradictory controls, that is, both activation and repression of the same gene target.

To determine whether fold-change detection in localization of NF-κB is universally implemented in NF-κB target genes, Wong et al. [176] used an I1-FFL model and single-cell live imaging to show that even genes with low levels of TNF-induced transcription induced by NF-κB are responsive to the fold-change in nuclear NF-κB [178]. Using live cell imaging, the authors tracked nuclear NF-κB after TNF-α stimulation and quantified the number of transcripts by RNA fluorescence *in situ* hybridization in the same cells. They found that the presence of TNF-α induces transcripts of low and high abundance target genes correlated with similar intensity of nuclear NF-κB fold change [178]. The I1-FFL model implements fold-change detection from competition for κB motif binding and shows that fold-change detection can be reproduced across the range of experimentally measured transcripts [178]. This study suggests that cells use the same mechanistic model for low and high levels of transcription genes and distinguish them by adopting different kinetic parameters. Thus, multiple biological mechanisms regulate transcriptional output while maintaining the robustness of NF-κB fold-change detection.

The studies summarized in this chapter show that NF-κB regulation at the single-cell level has been elucidated using mathematical models. A switch-like system and fold-change detection lead to robustness in NF-κB regulation. Linking these systems with aging will further elucidate the mechanistic structure of NF-κB signal transduction regulation in aging as a promising target for research.

## Discussion

In recent years, drug discovery and development based on systems biology have attracted increasing interest in the scientific and industrial fields [179,180]. Human disease models and multi-layered omics data can improve the success rate of drug development and predict the safety and efficacy of drugs in patients [181]. Systems biology is a powerful tool for deepening understanding of diseases, such as neurodegenerative diseases [182] and cancer [183]. Drugs related to NF-κB signaling have also been identified using systems biology [184].Pabon et al. [184] reported a network-centric drug discovery approach to predict drugs that inhibit NF-κB signaling and elucidate the network response. By integrating transcriptome analysis, machine learning, structural analysis, and live cell imaging, the authors showed that the maturation of multiprotein complexes required for IKK activation can be inhibited, thus effectively inhibiting NF-κB signaling. Oppelt et al. [185] used a mathematical model to identify factors — IKK and free cytoplasmic IκBα—that contribute to increased hepatotoxicity caused by the combination of TNF-α and the anti-inflammatory drug diclofenac. They not only proposed a new mathematical model of NF-ĸB but also used it to assess the safety of drugs.

Although cell fate is determined by the crosstalk of complex signaling networks, the priority in drug efficacy studies to date has been to confirm the efficacy of a single target. Because the phenotypic system of aging is a product of various signaling networks, a systems biology approach can allow us to view the entire system from a bird's eye view and extract its characteristics. This approach is expected to be applied not only to drug discovery but also to research on general commercial products for aging.

As introduced in this review, the application of mathematical models can elucidate complexes hidden under signal pathways. In the future, combining mathematical models of different signaling pathways for a certain disease or symptom will be important to comprehensively confirm the effects, which will lead to better understanding and treatment of the target disease. Methods are also being developed to accelerate the integration and utilization of the crosstalk of multiple models [121,186–189]. Software tools, such as MATLAB, Copasi [190], Berkeley Madonna [191], and CellDesigner [192,193], are used as modeling tools. For logical modeling (e.g. Boolean networks), tools such as BoolNet [194], MaBoSS [195], GINsim [196], and PyBoolNet [197] are widely used and are summarized by the CoLoMoTo consortium (http://www.colomoto.org/). Our group developed a Python-based ODE modeling and analysis tool for signaling systems called BioMASS [198], which allows multiple sets of parameters to be optimized simultaneously, generating multiple candidate parameters that describe the desired signaling dynamics. Using these tools allows us to integrate two or more separately developed models and perform parameter estimation to fit the experimental data of individual researchers.

## Conclusion

In addition to the classical roles of NF-κB, such as inflammation and response during immunity, the role of the NF-κB pathway should not be overlooked in terms of aging. In this review, we outlined the relationship between aging and NF-κB signaling, the contribution of multi-omics analysis to aging research, and the mathematical models indicating the relationship between NF-κB and aging. We summarized the dynamic properties of the NF-κB system and showed that NF-κB dynamics are meaningful for the regulation of downstream target genes. The integration of mathematical models and observation of cell dynamics, such as immunoprecipitation, live single-cell observation, and scRNA-seq analysis, is a powerful tool for deepening understanding of biological phenomena. A combination of both omics analysis and mathematical models will enable us to understand the whole system of aging; these research results are expected to be widely implemented in society in the near future.

## Data Availability

Data availability statements do not apply to this document as no new data were created or analyzed as part of this study.

## Abbreviations

ACE2: angiotensin-converting enzyme 2
CD: cluster of differentiation
ChIP: chromatin immunoprecipitation
CKII: casein kinase II
COVID-19: coronavirus disease 2019
CSE: cigarette smoke extract
GRN: gene regulatory network
GSEA: gene set enrichment analysis
H_2_O_2_: hydrogen peroxide
H3K27ac: H3 acetylation at lysine 27
H3K4me3: histone H3 trimethylation at lysine 4
HAGR: Human Aging Genomic Resources
HSCs: hematopoietic stem cells
I1-FFL: incoherent feedforward loop
IKK: inhibitor of κB kinase
IL: interleukin
IκB: inhibitor of κB
LPS: lipopolysaccharide
NEMO: NF-κB essential modulator
NF-κB: nuclear factor-κB
NIK: NF-κB inducing kinase
NK: natural killer
NMR: naked mole-rat
ODE: ordinary differential equation
P3CSK: Pam3-Cys-SK4
pHBEC: primary human bronchial epithelial cell
Poly(I:C): polyinosinic:polycytidylic acid
ROS: reactive oxygen species
RS: replicative stress
SAM: senescence-accelerated mouse
SAMP8: senescence-accelerated mouse prone 8
SARS-CoV-2: severe acute respiratory syndrome coronavirus disease 2
SASP: senescence-associated secretory phenotype
SA-β-gal: senescence-associated beta-galactosidase
scRNA-seq: single-cell RNA sequencing
STAT: signal transducer and activator of transcription
TAD: transactivation domain
TNF: tumor necrosis factor
UV: ultraviolet
WB: western blot
β-TrCP: beta-transduction repeat containing protein
β-TrCP: beta-transduction repeat containing protein
